# Clinical outcomes of increased focal amyloid uptake in individuals with subthreshold global amyloid levels

**DOI:** 10.3389/fnagi.2023.1124445

**Published:** 2023-03-02

**Authors:** Jaeho Kim, Yeong Sim Choe, Yuhyun Park, Yeshin Kim, Jun Pyo Kim, Hyemin Jang, Hee Jin Kim, Duk L. Na, Soo-Jin Cho, Seung Hwan Moon, Sang Won Seo

**Affiliations:** ^1^Department of Neurology, Samsung Medical Center, Sungkyunkwan University School of Medicine, Seoul, Republic of Korea; ^2^Neuroscience Center, Samsung Medical Center, Seoul, Republic of Korea; ^3^Samsung Alzheimer Research Center, Samsung Medical Center, Seoul, Republic of Korea; ^4^Department of Neurology, Dongtan Sacred Heart Hospital, Hallym University College of Medicine, Hwaseong-si, Gyeonggi-do, Republic of Korea; ^5^Department of Health Sciences and Technology, SAIHST, Samsung Medical Center, Sungkyunkwan University, Seoul, Republic of Korea; ^6^Department of Neurology, Kangwon National University College of Medicine, Chuncheon-si, Gangwon-do, Republic of Korea; ^7^Department of Radiology and Imaging Sciences, Indiana University School of Medicine, Indianapolis, IN, United States; ^8^Samsung Medical Center, Stem Cell and Regenerative Medicine Institute, Seoul, Republic of Korea; ^9^Department of Nuclear Medicine, Samsung Medical Center, Sungkyunkwan University School of Medicine, Seoul, Republic of Korea; ^10^Department of Clinical Research Design and Evaluation, SAIHST, Samsung Medical Center, Sungkyunkwan University, Seoul, Republic of Korea

**Keywords:** amyloid PET imaging, florbetaben, flutemetamol, Alzheimer’s disease, focal amyloid uptake

## Abstract

**Background:**

Although the standardized uptake value ratio (SUVR) method is objective and simple, cut-off optimization using global SUVR values may not reflect focal increased uptake in the cerebrum. The present study investigated clinical and neuroimaging characteristics according to focally increased β-amyloid (Aβ) uptake and global Aβ status.

**Methods:**

We recruited 968 participants with cognitive continuum. All participants underwent neuropsychological tests and 498 ^18^F-florbetaben (FBB) amyloid positron emission tomography (PET) and 470 ^18^F-flutemetamol (FMM) PET. Each PET scan was assessed in 10 regions (left and right frontal, lateral temporal, parietal, cingulate, and striatum) with focal-quantitative SUVR-based cutoff values for each region by using an iterative outlier approach.

**Results:**

A total of 62 (6.4%) subjects showed increased focal Aβ uptake with subthreshold global Aβ status [global (−) and focal (+) Aβ group, G(−)F(+) group]. The G(−)F(+) group showed worse performance in memory impairment (*p* < 0.001), global cognition (*p* = 0.009), greater hippocampal atrophy (*p* = 0.045), compared to those in the G(−)F(−). Participants with widespread Aβ involvement in the whole region [G(+)] showed worse neuropsychological (*p* < 0.001) and neuroimaging features (*p* < 0.001) than those with focal Aβ involvement G(−)F(+).

**Conclusion:**

Our findings suggest that individuals show distinctive clinical outcomes according to focally increased Aβ uptake and global Aβ status. Thus, researchers and clinicians should pay more attention to focal increased Aβ uptake in addition to global Aβ status.

## 1. Introduction

Cerebral β-amyloid (Aβ) deposition is one of the earliest recognizable pathological events in Alzheimer’s disease (AD; Bateman et al., 2012). Previous studies have shown that Aβ accumulation could occur for up to several decades before the onset of dementia symptoms. Thus, detecting the presence of Aβ is essential for the early diagnosis of AD (Sperling et al., 2014). Although ^11^C-Pittsburgh Compound-B (PiB) plays an important role in AD, ^18^F labeled positron emission tomography (PET) ligands such as ^18^F-florbetapir (FBP), ^18^F-florbetaben (FBB), and ^18^F-flutemetamol (FMM) have been developed and approved for clinical use because of their wider accessibility aided by the longer radioactive decay half-life compared to that for PiB (Hatashita et al., 2019; Cho et al., 2020b).

In the clinical trials targeting Aβ deposition in participants with MCI and early stage of dementia, the exclusion rate was over 70%, and this high exclusion rate was mostly related to Aβ positivity (+) on PET (Budd Haeberlein et al., 2022). In fact, previous studies showed that the frequency of Aβ (+) across the Alzheimer disease clinical spectrum was 57.0% for MCI and 85.7% for dementia using Aβ PET (Jansen et al., 2022). However, different cutoffs for Aβ (+) were used among clinical trials (Pemberton et al., 2022). A quantitative method for determining Aβ positivity (+) uses the standardized uptake value ratio (SUVR) in the global cortical-to-reference region. Although the SUVR method is simple and objective, the cut-off optimization using global SUVR values may not reflect focal increased uptake in the cerebrum. However, pathological studies suggested that there were epicenters in the deposition of Aβ (brain regions known to show early Aβ deposition) including the medial frontal, medial parietal, and lateral temporoparietal regions (Grothe et al., 2017; Palmqvist et al., 2017; Mattsson et al., 2019; Guo et al., 2021; Insel et al., 2021). Meanwhile, visual assessment is the only approved method for determining Aβ (+) on PET. Visual assessment emphasizes the importance of focally increased uptakes on PET. For example, even if only one region of 10 or 12 regions is positive by visual assessment, the final amyloid PET reading should be positive. However, in the case of focal amyloid uptake, the concordance rate and accuracy of visual assessment are relatively low (75–89%; Collij et al., 2021). Therefore, to assess focal amyloid uptake across datasets using different amyloid PET ligands consistently, standardized methods using focal Aβ uptake cut-offs are needed.

In the present study, we aimed to investigate the clinical and neuroimaging characteristics of participants with subthreshold levels of global Aβ who only showed increased uptakes of Aβ in focal regions. We hypothesized that participants with focally increased Aβ uptakes, even in subthreshold global Aβ levels, might show AD patterns of neuroimaging and neuropsychological features.

## 2. Methods

### 2.1. Participants

A total of 968 participants were recruited from the Memory Disorders Clinic of Samsung Medical Center (SMC) from August 1, 2015, to September 31, 2019. We selected participants aged 55 or older who were either cognitively unimpaired, or were clinically diagnosed with aMCI or Dementia. In order to recruit participants with cognitive continuum (Jack et al., 2018), we recruited participants with amnestic MCI who met the criteria proposed by Albert et al. (2011). Participants with dementia met the core clinical criteria of probable AD dementia proposed by the National Institute on Aging-Alzheimer’s Association (NIA-AA; McKhann et al., 2011). Participants with cognitively unimpaired (CU) met the following criteria: (a) Korean Mini-Mental State Examination (K-MMSE) score of ≥ 24 or more than-1.5 standard deviations (SD) from the age, sex, and education adjusted norms for an education period of < 9 years; (b) greater than the-1.0 SD from the age, sex, and education adjusted norms on the delayed recall of the Seoul Verbal Learning Test-Elderly’s version (SVLT-E); (c) greater than the-2.0 SD from the age, sex, and education adjusted norms on the Korean version of the Boston Naming Test (K-BNT), the Korean-Color Word Stroop test (K-CWST) color reading, and the Rey-Osterrieth Complex Figure Test (RCFT) copy; and (d) no history of other neurological disorders (Kang et al., 2019).

The screening was performed by trained clinicians and neuropsychologists. Brain magnetic resonance imaging (MRI) confirmed the absence of structural lesions including territorial cerebral infarction, brain tumors, hippocampal sclerosis, vascular malformation, and intracranial hemorrhage. All protocols were approved by the Institutional Review Board at each participating site and the participants signed written informed consent at the time of enrolment.

### 2.2. Amyloid pet data acquisition

A total of 968 participants underwent 498 ^18^F-florbetaben (FBB) and 470 ^18^F-flutemetamol (FMM) PET scanning at the SMC. Scanning was performed using a Discovery Ste. PET/computed tomography (CT) scanner (GE Medical Systems, Milwaukee, WI, United States) with a three-dimensional (3D) scanning mode that examined 47 slices of 3.3 mm thickness spanning the entire brain. Mean doses of 311.5 MBq FBB and 197.7 MBq FMM were injected before a 20-min emission PET scan with dynamic mode (4 × 5-min frames). The scans were performed 90 min after injection. 3D PET images were reconstructed in a 128 × 128 × 48 matrix with a 2 × 2 × 3.27 mm voxel size using the ordered-subsets expectation–maximization algorithm (iteration = 4 and subset = 20).

The PET images were co-registered to each MR image, which was normalized to a T1-weighted MNI-152 template using SPM8 in MATLAB 2014b (MathWorks, Natick, MA, United States). After standard space registration, we divided gray matter into 116 regions using the automated anatomical labeling (AAL) atlas (Tzourio-Mazoyer et al., 2002). We used the whole cerebellum in FBB and FMM as the regions of interest to the reference uptake ratio [which is identical to the standardized uptake value ratio (SUVR)].

### 2.3. Calculation of centiloid and definition of global amyloid positivity

To calculate the cut-off of global Aβ (+), we utilized Centiloid (CL) methods. Previously, we developed a direct comparison of FBB-FMM CL (dcCL) using head to head comparison of FBB and FMM dataset (20 Aβ PET negative (−) young controls, 16 Aβ (−) old controls, and Aβ positive (+) 20 participants with cognitive impairment; Cho et al., 2020a,c; Jang et al., 2021). Full processes are described in detail at previous studies (Klunk et al., 2015; Cho et al., 2020a). Briefly, the FBB-FMM CTX VOI was generated using SUVR parametric images (and the WC reference VOI) from the 20 typical ADCI patients (AD-CTX) as well as the 16 OCs (OC-CTX). To generate the FBB-FMM CTX VOI, the average OC-CTX image was subtracted from the average AD-CTX image. For direct comparison of the FBB-FMM conversion method, SUVR values for the FBB-FMM cortical target volume of interest (CTX VOI) were directly converted into Centiloid (CL) units using the dcCL method based on the CL conversion equation below (Klunk et al., 2015; Cho et al., 2020c):


$$\begin{array}{l} \mathrm{CL}=\\ 100\times \left({\mathrm{SUVR}}_{\mathrm{ind}}-{\mathrm{SUVR}}_{\mathrm{YC}-0}\right)/\left({\mathrm{SUVR}}_{\mathrm{ADCI}-100}-{\mathrm{SUVR}}_{\mathrm{YC}-0}\right) \end{array}$$

where SUVR_ind_ represents the individual SUVR values of all YC-0 and ADCI-100 participants, and SUVR_YC-0_ and SUVR_ADCI–100_ represent each group’s mean SUVR values. To obtain the dcCL cutoff value for amyloid positivity, we obtained the dcCL cutoff value for Aβ positivity, performing receiver operating characteristic (ROC) analysis with Aβ positivity based on the SUVR cutoff for each amyloid PET scan as the standard of truth (Jang et al., 2021). The optimal Centiloid cutoff value was set at 25.11.

### 2.4. Classification of participants according to the distributions of Aβ involvement

**Figure 1:**
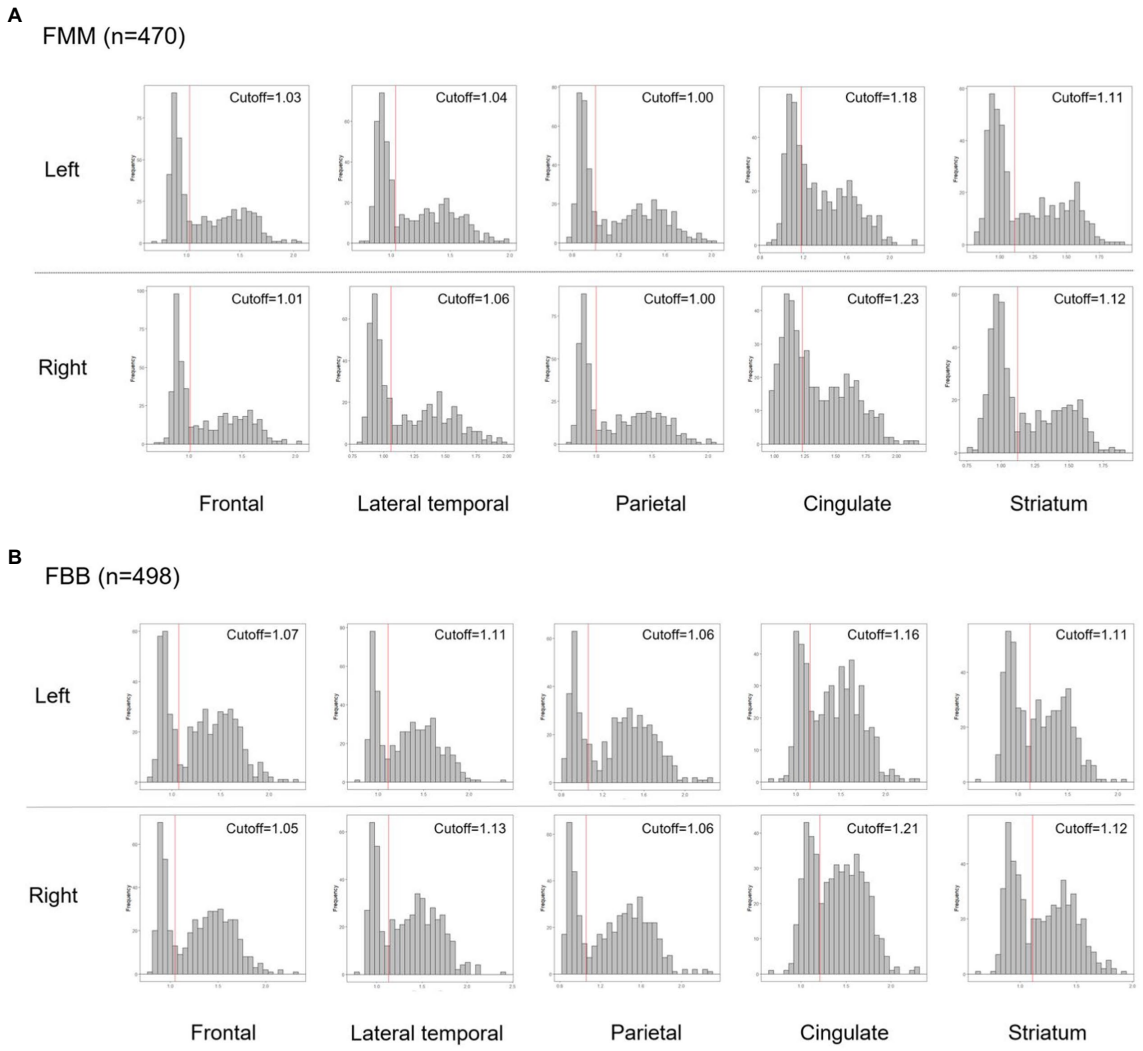
Histograms of the Aβ PET SUVR distribution in each Aβ ligand, FMM **(A)**, and FBB **(B)** for each region. Abbreviations: PET, positron emission tomography; FMM, Flutemetamol; FBB, Florbetaben; SUVR, standardized uptake value ratio.

We calculated focal-specific cut-off values of Aβ. In order to calculate the focal-specific cut-offs of Aβ (+), we grouped 28 VOIs into ten regions such as the frontal, lateral temporal, parietal, cingulate, and striatum in the left and right hemispheres. These regions were consistent with those widely used in other study groups. These regions also overlapped with those proposed by the FBB and FMM visual interpretation guidelines (Barthel et al., 2011; Salloway et al., 2017). In detail, each focal VOI was defined as the frontal (superior and middle frontal gyri, medial part of superior frontal gyrus, opercular part of inferior frontal gyrus, triangular part of inferior frontal gyrus, supplementary motor area, orbital part of superior, middle, and inferior orbital frontal gyri), lateral temporal (superior, middle, and inferior temporal gyri), parietal (superior and inferior parietal, supramarginal and angular gyri, and precuneus), cingulate gyri (anterior and posterior cingulate gyri), and striatal (caudate, putamen) areas. Since there was no method to calculate the focal CL, we used focal-specific cut-off values of Aβ SUVR in each Aβ ligand (Figure 1). We processed the iterative outlier method, generating an upper and lower bound SUVR. Subjects having values that were either higher than the upper inner-bound [third quartile + 1.5 interquartile range (IQR)] or less than the lower inner-bound (first quartile—1.5 IQR) were eliminated from the dataset during the iteration. Until all outliers had been eliminated, this procedure was repeated. A cutoff value for the final dataset was established by adding 2.5% of the highest SUVR in itself (Mormino et al., 2012). For the processing iterative outlier method, we used Aβ data obtained from 445 cognitively unimpaired (CU) participants aged over 55 years. Of the 445 participants, 220 and 225 subjects underwent FBB and FMM Aβ PET, respectively. Finally, we classified our participants as global (−) and global (+) according to the global cut-off value of Aβ dcCL. We also determined the presence of Aβ involvements in each of the 10 regions using the focal-specific cut-off values of Aβ SUVRs. Furthermore, we subclassified our global (−) participants as none or focal type, and global (+) participants as focal type or whole brain (W) type according to the number of focal Aβ involvements. Since there was the largest difference in global Aβ uptakes between the involved number of 9 regions and the involved number of 10 regions, the focal type was subclassified as increased Aβ uptake in 1–9 regions and the W type was subclassified as increased Aβ uptake in all 10 regions. Thus, we.

classified our participants into three groups: global (−) and focal (−) Aβ: [G(−)F(−)], global (−) and focal (+) Aβ: [G(−)F(+)], and whole brain (+) Aβ: [G(+)].

### 2.5. Acquisition of brain MRI and cortical thickness measurements

An Achieva 3.0 Tesla MRI scanner (Philips, Best, Netherlands) was used to acquire 3D T1 turbofield echo (TFE) MRI data from all participants using the following scanning parameters: sagittal slice thickness, 1.0 mm; over contiguous slices with 50% overlap; no gap; repetition time, 9.9 ms; echo time, 4.6 ms; flip angle, 8 degrees; and matrix size of 240 × 240 pixels reconstructed to 480 × 480 over a field of view of 240 mm.

To obtain local cortical thickness measurements for each subject, all T1 volume scans were processed using the CIVET pipeline (version 2.1.0) developed at the Montreal Neurological Institute for fully automated structural image analysis. In brief, using a linear transformation, native MRI images were registered to the MNI-152 template (Collins et al., 1994). The N3 algorithm was used to correct the intensity nonuniformity caused by the inhomogeneities in the magnetic field. The tissue was then classified as white matter (WM), gray matter (GM), cerebrospinal fluid (CSF), and background (BG) based on the T1-weighed images. The brain was split into the left and right hemispheres for surface extraction. The surfaces of the inner and outer cortices were automatically extracted using the constrained Laplacian-based automated segmentation with proximity algorithm. The inner and outer surfaces had the same numbers of vertices and there was a close correspondence between the counterpart vertices of the inner and outer cortical surfaces. Cortical thickness was defined as the Euclidean distance between the linked vertices of the inner and outer surfaces, with 40,962 vertices in each hemisphere in the native space.

Cortical thickness values were calculated in native brain rather than Talairach spaces because of the limitations of linear stereotaxic normalization. We defined intracranial volume (ICV) as the total volume of GM, WM, and cerebrospinal fluid in the native space considering of voxel dimension. Brain masks were generated using the functional MRI of the brain (FMRIB) software library (FSL) bet algorithm. Since cortical surface models were extracted from MRI volumes transformed into stereotaxic space, cortical thickness was measured in the native space by applying an inverse transformation matrix to the cortical surfaces and reconstructing them in the native space. We calculated mean cortical thickness in the AD-specific ROI including entorhinal cortex, parahippocampus, inferior parietal lobe, pars opercularis, pars orbitalis, pars triangularis, inferior temporal lobe, temporal pole, precuneus, supramarginal gyrus, superior parietal lobe, and superior frontal lobe (Parker et al., 2020).

To measure the hippocampal volume (HV), we used an automated hippocampus segmentation method using a graph cut algorithm combined with atlas-based segmentation and morphological opening, as described previously (Kwak et al., 2013).

### 2.6. Statistical analysis

To compare the demographic data, we used chi-square tests for categorical variables and analysis of variance followed by Tukey’s *post hoc* analysis to compare the continuous variables among the three groups. To compare the clinical and neuroimaging data, we used analysis of covariance after controlling for age followed by Tukey’s *post hoc* analysis to compare the continuous variables among the three groups. Since the G(−)F(+) group has a relatively small sample size, we also performed Mann–Whitney test to compare neuroimaging and neuropsychological features between the G(−)F(−) and the G(−)F(+) groups. Jonckheere-Terpstra tests were used to analyze *p*-values for trends within group differences. All analyses were performed using IBM SPSS Statistics for Windows, version 20.0 (IBM Corp.).

To evaluate the cortical thickness analyses of MRI data from the participants, we used a MATLAB-based toolbox.^1^

## 3. Results

### 3.1. Clinical characteristics of our participants

Table 1 presents the clinical characteristics of the participants. This study included 228 (23.6%) participants with CU, 400 (41.3%) with aMCI, and 340 (35.1%) with clinically diagnosed dementia. The mean age was 71.6 ± 8.2 years, 536 of 968 (55.4%) were female, and the mean years of education was 11.7 ± 4.9 years. The frequencies of G (+) were 20.6% in CU, 56.8% in aMCI, and 84.7% in dementia.

**Table 1:** Demographics of the study participants (total).

	All participants
No.	968
Age, mean ± SD, years	71.6 ± 8.2
Female sex	536 (55.4)
Education, mean ± SD, years	11.7 ± 4.9
*APOE4* carrier	387 (43.1)
K-MMSE	24.2 ± 5.4

**Figure 2:**
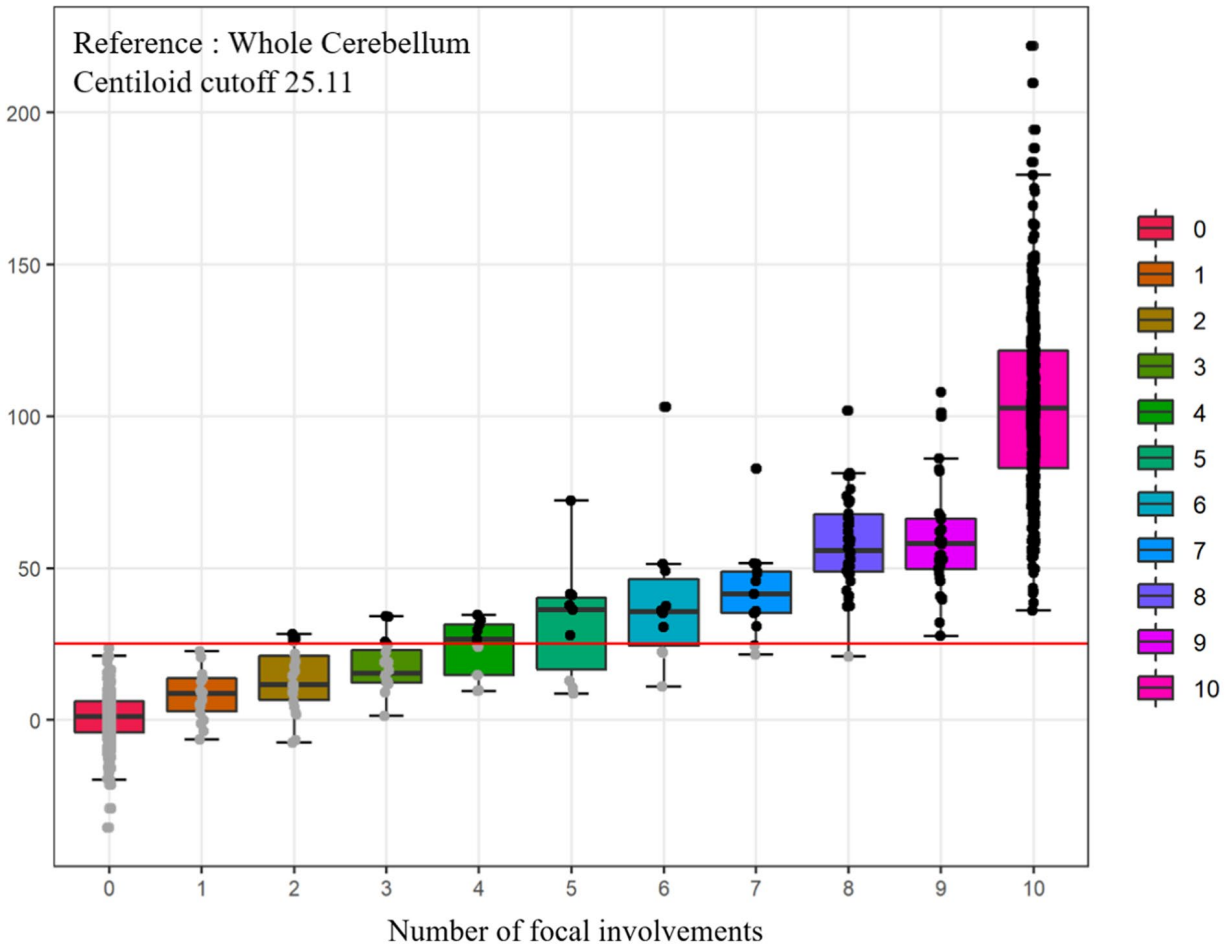
Comparisons of Centiloid values according to numbers of focal amyloid PET involvement. Total *n* = 968, FBB (*n* = 498, ref. WC), FMM (*n* = 470, ref. WC). PET, positron emission tomography; WC, whole cerebellum.

In this study, 35.5% of our participants were in the G(−)F(−) group, 6.4% in the G(−)F(+) group, 58.1% in the G(+) group (Table 2). The mean age was highest in the G(−)F(+) group (74.9 ± 6.6 years), followed G(+) group (71.6 ± 8.3 years), and G(−)F(−) group (70.9 ± 8.1 years). The frequency of apolipoprotein E (APOE) e4 carriers was the highest in the G(+) group (60.5%), followed by the G(−)F(+) group (32.7%), and G(−)F(−) group (15.8%). As the number of involved regions increased, the Centiloid value also increased (Figure 2). Amyloid PET results showed that participants with 1–4 focal involvements were mainly included in the G(−)F(+) group.

**Table 2:** Demographics of the study participants by group.

Group	G(−)F(−)	G(−)F(+)	G(+)	*p* Value
No.	344	62	562	
Age, mean ± SD, years	70.9 ± 8.1	74.9 ± 6.6^*^	71.6 ± 8.3^†^	0.002
Female sex	174 (50.6)	32 (51.6)	330 (58.7)^*^	0.047
Education, mean ± SD, years	12.0 ± 4.9	11.5 ± 5.4	11.5 ± 4.8	0.227
Group (CU/aMCI/dementia)	165/141/38	16/32/14	47/227/288	
*APOE4* carrier	50 (15.8)	18 (32.7)^*^	319 (60.5)^*^^,^^†^	<0.001
VA+	14 (4.1)	8 (12.9)^*^	523 (93.1)^*^^,^^†^	<0.001

VA, visual assessment.

* *p* < 0.05 group vs. G(−)F(−).

† *p* < 0.05 group vs. G(−)F(+).

**Figure 3:**
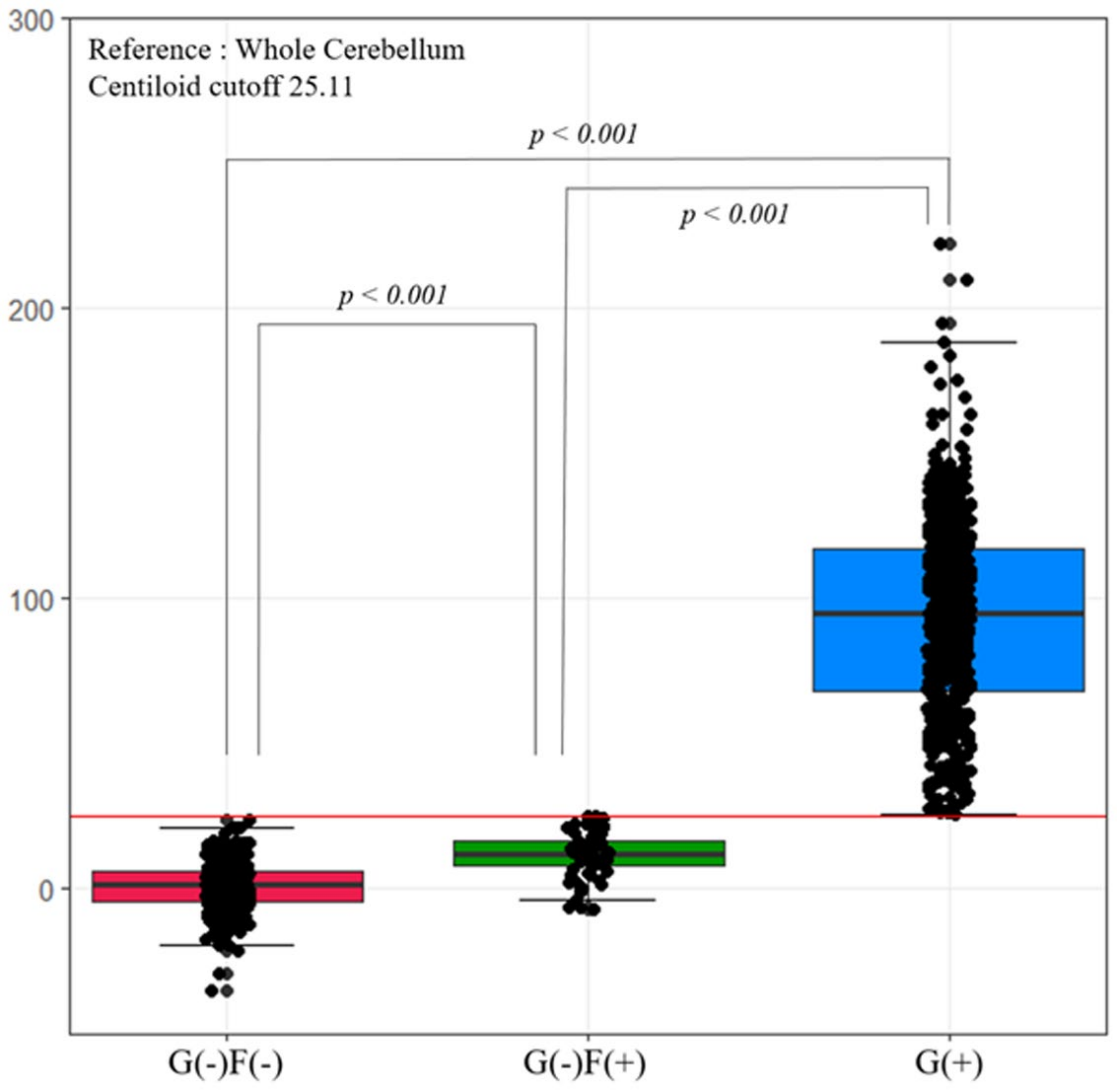
Centiloid values according to group. The Centiloid value of the G(−)F(−) and G(−)F(+) groups are lower than the cutoff values. Each group has an order of Centiliod value, which tends to increase from group G(−)F(−) to G(+). F, focal-type amyloid accumulation; G, global standardized uptake value ratio; W, whole region type amyloid accumulation.

Figure 3 shows the Centiloid values in each group. Each group had an order of Centiloid value and tended to increase from group G(−)F(−) to G(+).

**Figure 4:**
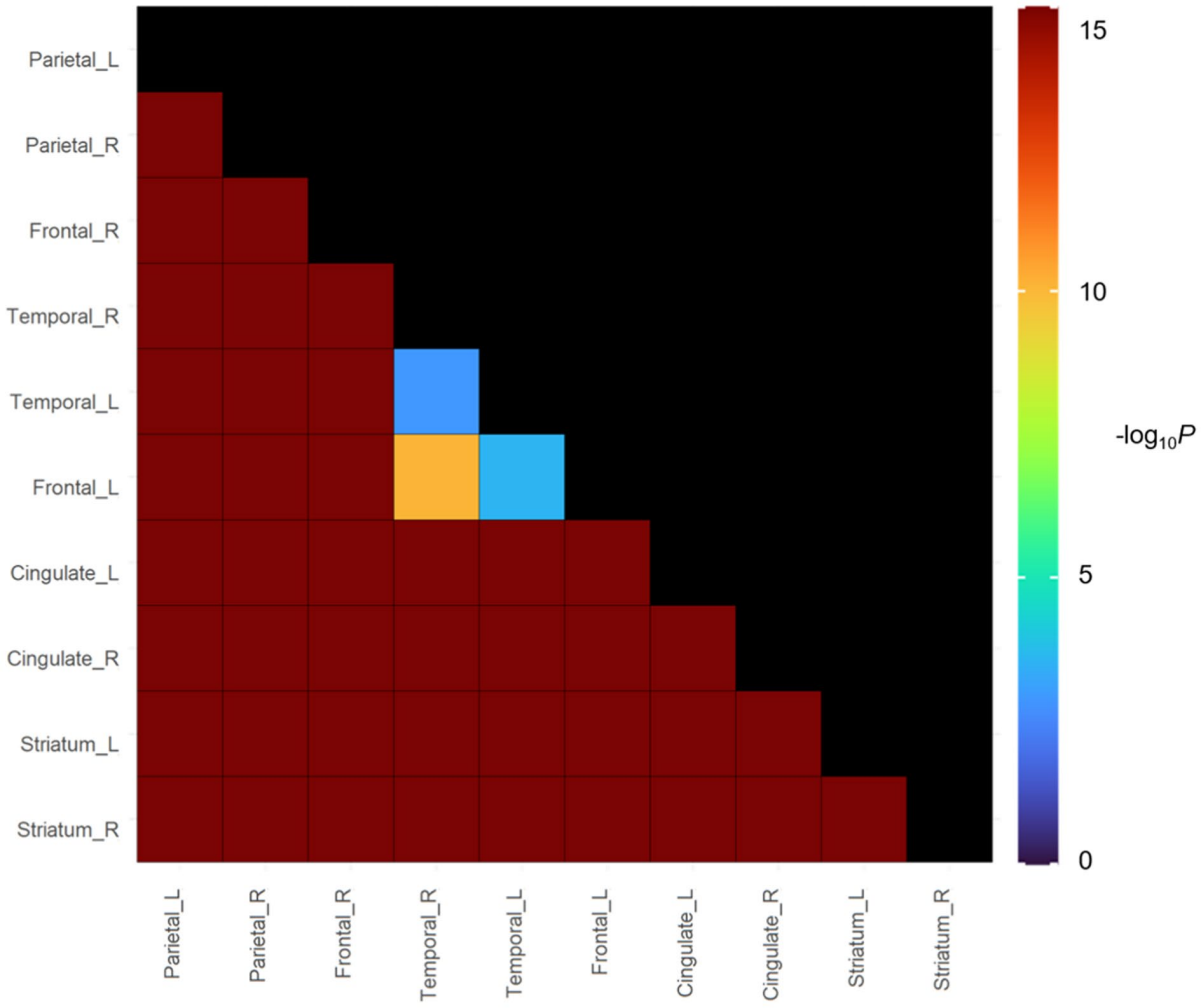
*In vivo* Spreading Order of focal amyloid PET group in cognitive continuum. Color bars represent logarithmic scale of *p* value (−log_10_).

In groups with focal involvements, amyloid accumulation was most frequently observed in the parietal lobe (38.8%), followed by the frontal lobe (32.5%), temporal lobe (32.4%), cingulate gyrus (26.1%) and striatum (13.0%). Bootstrapping with 1,000 resamples showed that the involvement frequencies of amyloid in the parietal, frontal, and temporal lobes were higher than those in the cingulate gyrus and striatum (Figure 4).

### 3.2. Comparisons of neuroimaging features among each group

**Figure 5:**
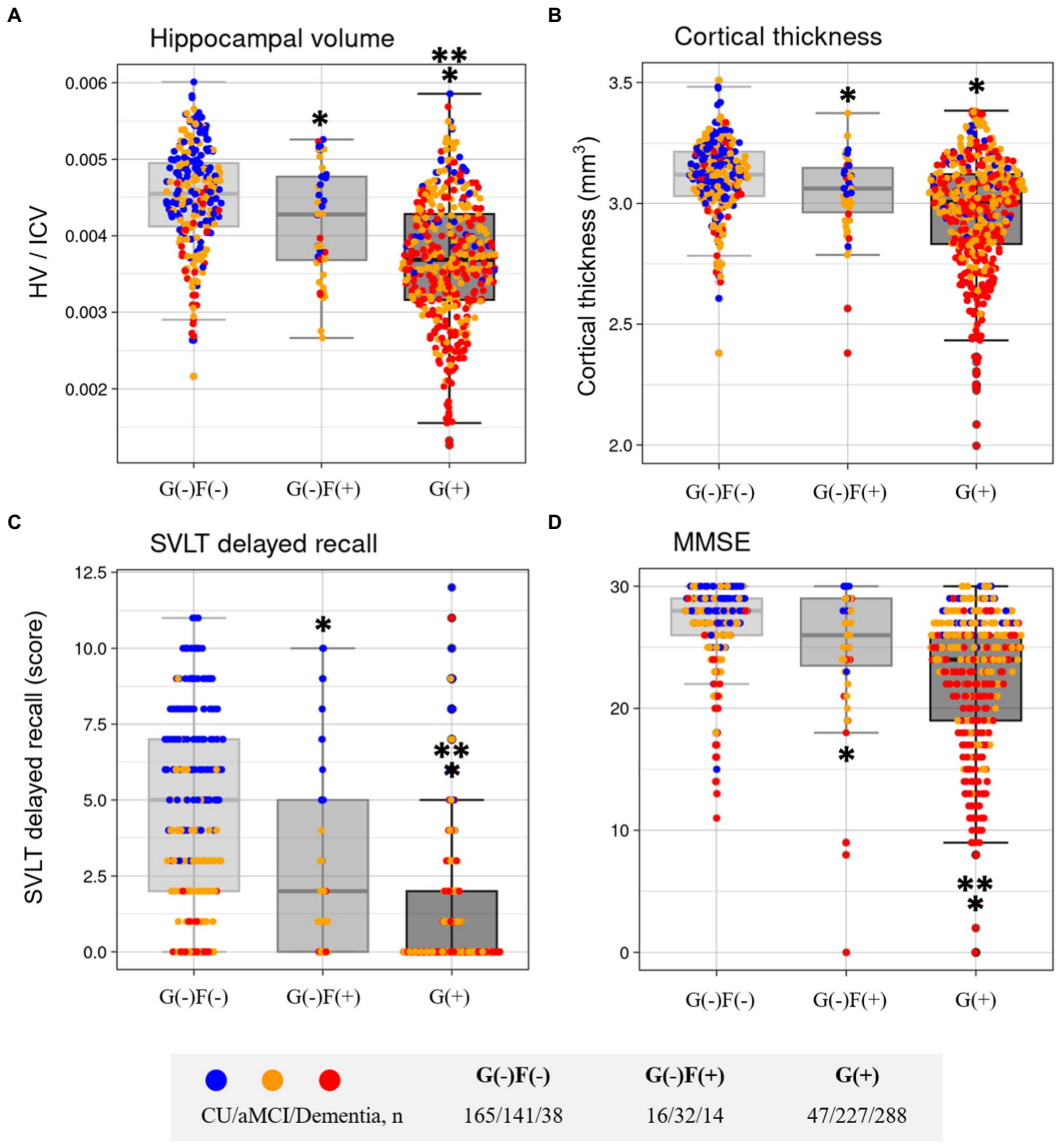
Comparisons of characteristics according to group in Hippocampal volume **(A)**, Cortical thickness **(B)**, SVLT delayed recall **(C)**, and MMSE **(D)**. F, focal type amyloid accumulation; G, global standardized uptake value ratio; HV, Hippocampal volume; ICV, intracranial volume; W, whole region type amyloid accumulation; SVLT, Seoul Verbal Learning Test-Elderly’s version; MMSE, Mini-Mental State Examination. ^*^p < 0.05 group vs. G(−)F(−). ^**^p < 0.05 group vs. G(−)F(+).

The neuroimaging characteristics of each amyloid involvement group are shown in Figure 5. The G(+) group showed a lower hippocampal volume (HV) than that in the G(−)F(+) group (*p* < 0.001) and G(−)F(−) group (*p* < 0.001). The HV was lower in the G(−)F(+) group than in the G(−)F(−) group (*p* = 0.045).

The widespread Aβ group (G(+)) showed decreased mean cortical thickness in the AD-specific ROI compared with G(−)F(−) (*p* < 0.001; Figure 5). The G(−)F(+) also showed decreased mean cortical thickness in the AD-specific ROI compared with G(−)F(−) (*p* = 0.034).

### 3.3. Comparison of neuropsychological features in each group

The neuropsychological and neuroimaging characteristics of each amyloid involvement group are shown in Figure 5. Compared to the G(−)F(−) group, the G(−)F(+) group showed lower SVLT (*p* < 0.001) and MMSE (*p* = 0.009) scores. The G(+) group showed lower performance in the SVLT (*p* < 0.001) and MMSE (*p* < 0.001) compared to that in the G(−)F(−) group. The SVLT and MMSE scores were lower in the G(+) group than in the G(−)F(+) group (*p* < 0.001, *p* = 0.004). The linear trend test showed a significant association between the severity of Aβ deposition [G(−)F(−), G(−)F(+), and G(+)] and cognitive function (*p* for trend < 0.001; Figure 3).

### 3.4. Sensitivity analysis

For sensitivity analysis, we applied 20 CL and 40 CL for alternative cut-off values. When the cut-off values were set to 20 CL, the number of participants were 344 in the G(−)F(−), 46 in the G(−)F(+), and 577 in the G(+) groups. There were no differences in HV (*p* = 0.473), mean cortical thickness (*p* = 0.893), SVLT (*p* = 0.078), and MMSE (*p* = 0.556) between the G(−)F(−) and G(−)F(+) groups. When the cut-off values were set to 40 CL, the number of participants were 346 in the G(−)F(−), 93 in the G(−)F(+), and 529 in the G(+) groups. The G(−)F(+) group showed lower HV (*p* = 0.004) compared to the G(−)F(−) group. The G(−)F(+) group also showed lower performance in the SVLT (*p* < 0.001) and MMSE (*p* < 0.001) compared to that in the G(−)F(−) group.

We also determined whether our findings might be driven by focally increased Aβ uptakes rather than relatively higher subthreshold dcCL levels *per se*. Based on the distribution of dcCL levels in the G(−)F(−) and G(−)F(+) groups, we classified our participants into negative (dcCL < 10), subthreshold (10 ≤ dcCL < 25.11), and positive (dcCL > 25.11) groups. We found that there were no differences in HV (*p* = 0.676), cortical thickness (*p* = 0.293), SVLT (*p* = 0.334), and MMSE (*p* = 0.222) between the negative and subthreshold groups. We also found that the AIC of the F/G grouping showed a lower value in HV(−8369.686 vs. –8365.577), cortical thickness(−300.9323 vs. –295.8582), SVLT (4414.42 vs. 4433.65), and MMSE (4703.362 vs. 4711.426) than the AIC of grouping based on dcCL levels, suggesting that the F/G grouping showed a better fit than grouping based on dcCL levels. Since the G(−)F(+) group has a relatively small sample size, we also performed Mann–Whitney test. There were differences in HV (*p* = 0.03), mean cortical thickness in the AD-specific ROI (*p* = 0.018), SVLT score (*p* < 0.001), and MMSE (*p* = 0.021) between the G(−)F(−) and G(−)F(+) groups.

In non-demented individuals, there were differences in HV (*p* = 0.032) and SVLT score (*p* < 0.001), but not mean cortical thickness in the AD-specific ROI (*p* = 0.185) and MMSE (*p* = 0.324) between the G(−)F(−) and the G(−)F(+) groups.

## 4. Discussion

We characterized increased focal Aβ uptake with subthreshold global Aβ levels based on ^18^F labeled PET (FBB and FMM) scan findings in a relatively large cohort of carefully phenotyped participants, using standardized neuropsychological and neuroimaging features. The major findings of our study are as follows: First, 6.4% of our participants showed increased focal Aβ uptake with subthreshold global Aβ status [G(−)F(+) group]. Second, participants with focally increased Aβ uptake showed changes in AD, including memory impairment, hippocampal atrophy, and cortical thickness in the AD-specific ROI, even with subthreshold global amyloid levels. Together, our findings suggest distinctive clinical outcomes according to focally increased Aβ uptake even with global subthreshold levels.

We used frontal, lateral temporal, parietal, cingulate, and striatum as focal measurements. These areas are largely consistent with the areas in protocols provided by the two companies that developed amyloid PET ligands (FBB and FMM; Barthel et al., 2011; Salloway et al., 2017). Recently, the importance of the striatum region in AD has been increasing (Kim et al., 2020), so this was also included in focal measurement. The cutoff values in cortical target regions seemed to be higher in the FBB than in the FMM, which is consistent with the results of our previous studies showing that the cortical SUVR was higher in the FBB than in the FMM.

Our first major finding was that 6.4% of our participants showed increased focal Aβ uptake with subthreshold global Aβ status [G(−)F(+) group]. Most participants with 3–4 or fewer involved regions were included in the G(−)F(+) group. Our findings are consistent with those of previous *in vivo* Aβ PET staging studies. A focal frequency-based staging study showed that many cases with lower stages (81% in stage I and 25% stage II) were missed by semiquantitative classification approaches based on suprathreshold global SUVR, even at a relatively lenient cutoff (Grothe et al., 2017). Consistent with previous studies, our findings were explained by those of imaging-pathology correlation studies showing that current cut-off optimization methods using several categorization approaches have limitations in detecting mild to moderate degrees of neuritic plaques in the brain. Another finding of rare striatal involvement in the G(−)F(+) group was also explained by the striatum-based staging from our group and another group showing that the striatal involvement of Aβ occurs only after cortical involvement (Hanseeuw et al., 2018). Although the previous study showed that visual assessment of FMM PET images might detect early amyloid pathology (Collij et al., 2021), in the present study, most participants in the G(−)F(+) group (87.1%) were classified as Aβ-negative by using visual assessment.

In the present study, nearly 75% of participants in the G(−)F(+) group did not demonstrate clinically diagnosed dementia. The G(−)F(+) group also showed an older age and higher frequency of *APOE4* carriers relative to those in both the G(−)F(−) and widespread G(+) groups. Considering that *APOE4* brings the start of amyloid pathology earlier, our findings suggest that the increased focal uptake in the G(−)F(+) group might be mainly driven by combination of older age and *APOE4*. Alternatively, as current Aβ PET ligands are limited in detecting the early state of Aβ accumulation, including diffuse plaques and sparse-to-moderate degrees of neuritic plaques (Jack et al., 2013), amyloid accumulation may appear in focal form amyloid PET testing, though amyloid deposition is actually positive. In an imaging-pathological correlation study, cases who seemed to have increased focal uptakes on PET usually showed increased uptakes in more extensive regions at autopsy (Villeneuve et al., 2015).

Our findings of last Aβ involvement in the striatum are consistent with Aβ PET findings (Grothe et al., 2017; Hanseeuw et al., 2018) and Aβ Thal pathologic staging (Thal et al., 2015). However, there were some discrepancies in the order of Aβ involvements within cortical regions among Aβ PET studies (Grothe et al., 2017; Mattsson et al., 2019; Collij et al., 2020). Specifically, other studies showed that cingulate or posterior cingulate is the initially involved region while our study did not show the results. However, our findings are consistent with Aβ pathological staging. Specifically, Aβ Thal pathologic staging showed that limbic involvements (Phase 2) were followed by neocortical involvements (Phase 1). Braak Aβ staging also showed that involvement in the cingulate was observed in stage B. Thus, further longitudinal studies with Aβ PET should be needed to investigate the order of Aβ involvements with cortical regions.

Our second major finding was that participants with focally increased Aβ uptake demonstrated changes in AD, including memory impairments, hippocampal atrophy, and cortical thickness in the AD-specific ROI even with subthreshold global amyloid levels. As these neuropsychological and neuroimaging features are widely known as changes associated with AD (Jack et al., 2000; Jahn, 2013), our findings suggest that G(−)F(+) represents AD changes. Previous studies showed that increasing stages of amyloid positive participants were associated with worse cognitive function and developed clinical stages (Grothe et al., 2017; Mattsson et al., 2019; Collij et al., 2020). Furthermore, Landau et al. also showed that subthreshold amyloid deposition is accompanied with memory decline in Aβ negative older adults (Landau et al., 2018). Thus, our findings suggest that the focal amyloid-positive scan group, despite global SUVR negativity, showed signs of neurodegenerative changes in clinical and neuroimaging findings, which might support clinicians in decision-making with patients with focal amyloid involvement despite negative amyloid PET diagnosis. A recent study also showed that participants with focal Aβ stage are relatively free from tau pathology and related to cognitive decline, proposing an ideal target for anti-amyloid treatments (Ozlen et al., 2022).

When we used the cut-off value (40 CL) defined by “elevated Aβ,” our findings remained significant. However, when we used the cut-off value (20 CL) which corresponded to “at least moderate Aβ plaque density” on neuropathology (Rafii et al., 2022), the statistical significance of our findings disappeared. Previously, the cut-off values of 26 CL represented high correlation with positive visual read (Amadoru et al., 2020) and optimal prediction of progression to dementia (Hanseeuw et al., 2021). Therefore, our findings using the cut-off value of 25.11 CL might have significant implications in clinical studies, especially in light of expected disease modifying therapies using the cut-off value of “elevated Aβ.”

The strengths of our study are the relatively large sample size of ^18^F-labeled amyloid PET scans in participants. However, our study has some limitations. First, our study used a cross-sectional design; therefore, we did not analyze the cognitive trajectory of participants in each group. Further longitudinal studies are needed to confirm whether focal amyloid-positive participants show different clinical courses. Second, pathologic verification was lacking and pathologic Aβ burdens should be used in further studies to validate the results. Third, there is also a wide range of sizes of the ROIs. A huge ROI result in more smoothed data relative to smaller ROIs like the cingulate. The smoothness of the data is highly likely to impact where the SUVR cutoff falls using an iterative outlier removal approach. Fourth, although the iterative outlier removal method is an established method for PET threshold derivation, it is highly dependent on how the data is distributed. Especially, the cingulate has the highest dynamic range in SUVR of all ROIs and naturally an iterative outlier removal approach is likely to be biased higher as a result. Fifth, we did not apply any correction method for partial volume effect, which might reduce global dcCL values in the G-F+ group. However, the original Klunk’s CL methods did not correct partial volume effect. Sixth, striatum ROI is not a validated region for FBB. However, this argument might be mitigated by our head-to-head comparison study of FMM and FBB showing that there was high correlation in striatal SUVR between two ligands (*R*^2^ = 0.95), although the striatal SUVR ratio was higher in FMM than in FBB (*p* < 0.001; Cho et al., 2020b). Finally, the standard for the presence or absence of amyloid PET scans in each brain region has not yet been established. While we defined amyloid positivity in each region based on the focal PET SUVR cutoff, further focal pathologic verifications are needed for cutoff values.

## 5. Conclusion

Clinically diagnosed possible or probable AD related MCI and dementia patients might be classified as the non-AD group when their global Aβ uptakes are within subthreshold levels. However, our findings revealed that participants with focally increased Aβ uptakes, even in subthreshold global Aβ levels, showed AD patterns of neuroimaging and neuropsychological features. Thus, researchers and clinicians should pay more attention to focally increased Aβ uptakes even in subthreshold global Aβ levels.

## Data availability statement

The raw data supporting the conclusions of this article will be made available by the authors, without undue reservation.

## Ethics statement

The studies involving human participants were reviewed and approved by Institutional Review Board, Samsung Medical Center. The patients/participants provided their written informed consent to participate in this study.

## Author contributions

JaK: study concept and design, acquisition, analysis and interpretation of data. YC and YP: analysis of data. YK, JuK, HJ, HK, and DN: acquisition of data, critical revision of manuscript. S-JC: critical revision of manuscript. SM and SS: study concept and design, acquisition, analysis and interpretation of data, critical revision of manuscript for intellectual content. All authors contributed to the article and approved the submitted version.

## Funding

This research was supported by a grant from the Korea Health Technology R&D Project through the Korea Health Industry Development Institute (KHIDI), funded by the Ministry of Health & Welfare and Ministry of Science and ICT, Republic of Korea (grant number HU20C0111, HU22C0170), the National Research Foundation of Korea (NRF) grant funded by the Korea government (MSIT) (NRF-2019R1A5A2027340, NRF-2022R1C1C1010435), Future Medicine 20*30 Project of the Samsung Medical Center [#SMX1230081], the “National Institute of Health” research project (2021-ER1006-02), the Hallym University Medical Center Research Fund, and partly supported by Institute of Information & communications Technology Planning & Evaluation (IITP) grant funded by the Korea government (MSIT) (No.2021-0-02068, Artificial Intelligence Innovation Hub).

## Conflict of interest

The authors declare that the research was conducted in the absence of any commercial or financial relationships that could be construed as a potential conflict of interest.

## Publisher’s note

All claims expressed in this article are solely those of the authors and do not necessarily represent those of their affiliated organizations, or those of the publisher, the editors and the reviewers. Any product that may be evaluated in this article, or claim that may be made by its manufacturer, is not guaranteed or endorsed by the publisher.

## Footnotes

^1^https://www.math.mcgill.ca/keith/surfstat/

## Abbreviations

Aβ: beta amyloid
D: diffuse-type amyloid accumulation
F: focal-type amyloid accumulation
G: global standardized uptake value ratio
MMSE: mini-mental state examination
PET: positron emission tomography
SMC: Samsung Medical Center
SUVR: standardized uptake value ratio
SVLT: Seoul Verbal Learning Test-Elderly’s version
WC: whole cerebellum

## References

[ref1] Albert M. S. Dekosky S. T. Dickson D. Dubois B. Feldman H. H. Fox N. C.. (2011). The diagnosis of mild cognitive impairment due to Alzheimer's disease: recommendations from the National Institute on Aging-Alzheimer's Association workgroups on diagnostic guidelines for Alzheimer's disease. Alzheimers Dement. 7, 270–279. doi: 10.1016/j.jalz.2011.03.008, PMID: 21514249 PMC3312027

[ref2] Amadoru S. Dore V. Mclean C. A. Hinton F. Shepherd C. E. Halliday G. M.. (2020). Comparison of amyloid PET measured in centiloid units with neuropathological findings in Alzheimer's disease. Alzheimers Res. Ther. 12:22. doi: 10.1186/s13195-020-00587-5, PMID: 32131891 PMC7057642

[ref3] Barthel H. Gertz H. J. Dresel S. Peters O. Bartenstein P. Buerger K.. (2011). Cerebral amyloid-β PET with florbetaben (18F) in patients with Alzheimer's disease and healthy controls: a multicentre phase 2 diagnostic study. Lancet Neurol. 10, 424–435. doi: 10.1016/S1474-4422(11)70077-1, PMID: 21481640

[ref4] Bateman R. J. Xiong C. Benzinger T. L. Fagan A. M. Goate A. Fox N. C.. (2012). Clinical and biomarker changes in dominantly inherited Alzheimer's disease. N. Engl. J. Med. 367, 795–804. doi: 10.1056/NEJMoa1202753, PMID: 22784036 PMC3474597

[ref5] Budd Haeberlein S. Aisen P. S. Barkhof F. Chalkias S. Chen T. Cohen S.. (2022). Two randomized phase 3 studies of Aducanumab in early Alzheimer's disease. J. Prev Alzheimers Dis. 9, 197–210. doi: 10.14283/jpad.2022.30, PMID: 35542991

[ref6] Cho S. H. Choe Y. S. Kim H. J. Jang H. Kim Y. Kim S. E.. (2020a). A new Centiloid method for (18) F-florbetaben and (18) F-flutemetamol PET without conversion to PiB. Eur. J. Nucl. Med. Mol. Imaging 47, 1938–1948. doi: 10.1007/s00259-019-04596-x, PMID: 31834446

[ref7] Cho S. H. Choe Y. S. Kim Y. J. Kim H. J. Jang H. Kim Y.. (2020b). Head-to-head comparison of 18F-Florbetaben and 18F-Flutemetamol in the cortical and striatal regions. J. Alzheimers Dis. 76, 281–290. doi: 10.3233/JAD-200079, PMID: 32474468 PMC9711935

[ref8] Cho S. H. Choe Y. S. Park S. Kim Y. J. Kim H. J. Jang H.. (2020c). Appropriate reference region selection of (18) F-florbetaben and (18) F-flutemetamol beta-amyloid PET expressed in Centiloid. Sci. Rep. 10:14950. doi: 10.1038/s41598-020-70978-z, PMID: 32917930 PMC7486392

[ref9] Collij L. E. Heeman F. Salvadó G. Ingala S. Altomare D. De Wilde A.. (2020). Multitracer model for staging cortical amyloid deposition using PET imaging. Neurology 95, e1538–e1553. doi: 10.1212/WNL.0000000000010256 32675080 PMC7713745

[ref10] Collij L. E. Salvadó G. Shekari M. Lopes Alves I. Reimand J. Wink A. M.. (2021). Visual assessment of [(18) F] flutemetamol PET images can detect early amyloid pathology and grade its extent. Eur. J. Nucl. Med. Mol. Imaging 48, 2169–2182. doi: 10.1007/s00259-020-05174-2, PMID: 33615397 PMC8175297

[ref11] Collins D. L. Neelin P. Peters T. M. Evans A. C. (1994). Automatic 3D intersubject registration of MR volumetric data in standardized Talairach space. J. Comput. Assist. Tomogr. 18, 192–205. doi: 10.1097/00004728-199403000-00005, PMID: 8126267

[ref12] Grothe M. J. Barthel H. Sepulcre J. Dyrba M. Sabri O. Teipel S. J. (2017). *In vivo* staging of regional amyloid deposition. Neurology 89, 2031–2038. doi: 10.1212/WNL.0000000000004643, PMID: 29046362 PMC5711511

[ref13] Guo T. Landau S. M. Jagust W. J. (2021). Age, vascular disease, and Alzheimer's disease pathologies in amyloid negative elderly adults. Alzheimers Res. Ther. 13:174. doi: 10.1186/s13195-021-00913-5, PMID: 34654465 PMC8520216

[ref14] Hanseeuw B. J. Betensky R. A. Mormino E. C. Schultz A. P. Sepulcre J. Becker J. A.. (2018). PET staging of amyloidosis using striatum. Alzheimers Dement. 14, 1281–1292. doi: 10.1016/j.jalz.2018.04.011, PMID: 29792874 PMC6219621

[ref15] Hanseeuw B. J. Malotaux V. Dricot L. Quenon L. Sznajer Y. Cerman J.. (2021). Defining a Centiloid scale threshold predicting long-term progression to dementia in patients attending the memory clinic: an [(18) F] flutemetamol amyloid PET study. Eur. J. Nucl. Med. Mol. Imaging 48, 302–310. doi: 10.1007/s00259-020-04942-4, PMID: 32601802 PMC7835306

[ref16] Hatashita S. Wakebe D. Kikuchi Y. Ichijo A. (2019). Longitudinal assessment of Amyloid-β deposition by [18F]-Flutemetamol PET imaging compared with [11C]-PIB across the Spectrum of Alzheimer’s disease. Front. Aging Neurosci. 11:251. doi: 10.3389/fnagi.2019.00251 31572167 PMC6749067

[ref17] Insel P. S. Donohue M. C. Berron D. Hansson O. Mattsson-Carlgren N. (2021). Time between milestone events in the Alzheimer's disease amyloid cascade. NeuroImage 227:117676. doi: 10.1016/j.neuroimage.2020.117676, PMID: 33359337

[ref18] Jack C. R. Bennett D. A. Blennow K. Carrillo M. C. Dunn B. Haeberlein S. B.. (2018). NIA-AA research framework: toward a biological definition of Alzheimer's disease. Alzheimers Dement. 14, 535–562. doi: 10.1016/j.jalz.2018.02.018, PMID: 29653606 PMC5958625

[ref19] Jack C. R. Jr. Knopman D. S. Jagust W. J. Petersen R. C. Weiner M. W. Aisen P. S.. (2013). Tracking pathophysiological processes in Alzheimer's disease: an updated hypothetical model of dynamic biomarkers. Lancet Neurol. 12, 207–216. doi: 10.1016/S1474-4422(12)70291-0, PMID: 23332364 PMC3622225

[ref20] Jack C. R. Jr. Petersen R. C. Xu Y. Obrien P. C. Smith G. E. Ivnik R. J.. (2000). Rates of hippocampal atrophy correlate with change in clinical status in aging and AD. Neurology 55, 484–490. doi: 10.1212/WNL.55.4.484, PMID: 10953178 PMC2724764

[ref21] Jahn H. (2013). Memory loss in Alzheimer's disease. Dialogues Clin. Neurosci. 15, 445–454. doi: 10.31887/DCNS.2013.15.4/hjahn, PMID: 24459411 PMC3898682

[ref22] Jang H. Kim J. S. Lee H. J. Kim C. H. Na D. L. Kim H. J.. (2021). Performance of the plasma Aβ42/Aβ40 ratio, measured with a novel HPLC-MS/MS method, as a biomarker of amyloid PET status in a DPUK-KOREAN cohort. Alzheimers Res. Ther. 13:179. doi: 10.1186/s13195-021-00911-7, PMID: 34686209 PMC8540152

[ref23] Jansen W. J. Janssen O. Tijms B. M. Vos S. J. B. Ossenkoppele R. Visser P. J.. (2022). Prevalence estimates of Amyloid abnormality across the Alzheimer disease clinical Spectrum. JAMA Neurol. 79, 228–243. doi: 10.1001/jamaneurol.2021.5216, PMID: 35099509 PMC12138908

[ref24] Kang S. H. Park Y. H. Lee D. Kim J. P. (2019). The cortical neuroanatomy related to specific neuropsychological deficits in Alzheimer's continuum. Dement. Neurocogn. Disord. 18, 77–95. doi: 10.12779/dnd.2019.18.3.77 31681443 PMC6819670

[ref25] Kim S. E. Lee B. Park S. Cho S. H. Kim S. J. Kim Y.. (2020). Clinical significance of focal ß-amyloid deposition measured by (18) F-flutemetamol PET. Alzheimers Res. Ther. 12:6. doi: 10.1186/s13195-019-0577-x, PMID: 31901233 PMC6942396

[ref26] Klunk W. E. Koeppe R. A. Price J. C. Benzinger T. L. Devous M. D. Sr. Jagust W. J.. (2015). The Centiloid project: standardizing quantitative amyloid plaque estimation by PET. Alzheimers Dement. 11:1. doi: 10.1016/j.jalz.2014.07.003 25443857 PMC4300247

[ref27] Kwak K. Yoon U. Lee D. K. Kim G. H. Seo S. W. Na D. L.. (2013). Fully-automated approach to hippocampus segmentation using a graph-cuts algorithm combined with atlas-based segmentation and morphological opening. Magn. Reson. Imaging 31, 1190–1196. doi: 10.1016/j.mri.2013.04.008, PMID: 23684964

[ref28] Landau S. M. Horng A. Jagust W. J. (2018). Memory decline accompanies subthreshold amyloid accumulation. Neurology 90, e1452–e1460. doi: 10.1212/WNL.0000000000005354, PMID: 29572282 PMC5921038

[ref29] Mattsson N. Palmqvist S. Stomrud E. Vogel J. Hansson O. (2019). Staging β-Amyloid pathology with Amyloid positron emission tomography. JAMA Neurol. 76, 1319–1329. doi: 10.1001/jamaneurol.2019.2214, PMID: 31314895 PMC6646987

[ref30] Mckhann G. M. Knopman D. S. Chertkow H. Hyman B. T. Jack C. R. Jr. Kawas C. H.. (2011). The diagnosis of dementia due to Alzheimer's disease: recommendations from the National Institute on Aging-Alzheimer's Association workgroups on diagnostic guidelines for Alzheimer's disease. Alzheimers Dement. 7, 263–269. doi: 10.1016/j.jalz.2011.03.005, PMID: 21514250 PMC3312024

[ref31] Mormino E. C. Brandel M. G. Madison C. M. Rabinovici G. D. Marks S. Baker S. L.. (2012). Not quite PIB-positive, not quite PIB-negative: slight PIB elevations in elderly normal control subjects are biologically relevant. NeuroImage 59, 1152–1160. doi: 10.1016/j.neuroimage.2011.07.098, PMID: 21884802 PMC3230690

[ref32] Ozlen H. Pichet Binette A. Kobe T. Meyer P. F. Gonneaud J. St-Onge F.. (2022). Spatial extent of Amyloid-beta levels and associations with tau-PET and cognition. JAMA Neurol. 79, 1025–1035. doi: 10.1001/jamaneurol.2022.2442, PMID: 35994280 PMC9396472

[ref33] Palmqvist S. Schöll M. Strandberg O. Mattsson N. Stomrud E. Zetterberg H.. (2017). Earliest accumulation of β-amyloid occurs within the default-mode network and concurrently affects brain connectivity. Nat. Commun. 8:1214. doi: 10.1038/s41467-017-01150-x, PMID: 29089479 PMC5663717

[ref34] Parker T. D. Cash D. M. Lane C. A. Lu K. Malone I. B. Nicholas J. M.. (2020). Amyloid β influences the relationship between cortical thickness and vascular load. Alzheimers Dement. (Amst) 12:e12022. doi: 10.1002/dad2.12022 32313829 PMC7163924

[ref35] Pemberton H. G. Collij L. E. Heeman F. Bollack A. Shekari M. Salvado G.. (2022). Quantification of amyloid PET for future clinical use: a state-of-the-art review. Eur. J. Nucl. Med. Mol. Imaging 49, 3508–3528. doi: 10.1007/s00259-022-05784-y, PMID: 35389071 PMC9308604

[ref36] Rafii M. S. Sperling R. A. Donohue M. C. Zhou J. Roberts C. Irizarry M. C.. (2022). The AHEAD 3-45 Study: design of a prevention trial for Alzheimer's disease. Alzheimers Dement. doi: 10.1002/alz.12748, PMID: 35971310 PMC9929028

[ref37] Salloway S. Gamez J. E. Singh U. Sadowsky C. H. Villena T. Sabbagh M. N.. (2017). Performance of [(18) F] flutemetamol amyloid imaging against the neuritic plaque component of CERAD and the current (2012) NIA-AA recommendations for the neuropathologic diagnosis of Alzheimer's disease. Alzheimers Dement. (Amsterdam, Netherlands) 9, 25–34. doi: 10.1016/j.dadm.2017.06.001, PMID: 28795133 PMC5536824

[ref38] Sperling R. Mormino E. Johnson K. (2014). The evolution of preclinical Alzheimer's disease: implications for prevention trials. Neuron 84, 608–622. doi: 10.1016/j.neuron.2014.10.038, PMID: 25442939 PMC4285623

[ref39] Thal D. R. Beach T. G. Zanette M. Heurling K. Chakrabarty A. Ismail A.. (2015). [18F] flutemetamol amyloid positron emission tomography in preclinical and symptomatic Alzheimer's disease: specific detection of advanced phases of amyloid-β pathology. Alzheimers Dement. 11, 975–985. doi: 10.1016/j.jalz.2015.05.018, PMID: 26141264

[ref40] Tzourio-Mazoyer N. Landeau B. Papathanassiou D. Crivello F. Etard O. Delcroix N.. (2002). Automated anatomical labeling of activations in SPM using a macroscopic anatomical parcellation of the MNI MRI single-subject brain. NeuroImage 15, 273–289. doi: 10.1006/nimg.2001.0978, PMID: 11771995

[ref41] Villeneuve S. Rabinovici G. D. Cohn-Sheehy B. I. Madison C. Ayakta N. Ghosh P. M.. (2015). Existing Pittsburgh compound-B positron emission tomography thresholds are too high: statistical and pathological evaluation. Brain 138, 2020–2033. doi: 10.1093/brain/awv112, PMID: 25953778 PMC4806716

